# Electrochemical sensor based on multi-walled carbon nanotubes and two-dimensional zeolitic imidazolate framework nanosheets: Application in determining dacarbazine

**DOI:** 10.5599/admet.2617

**Published:** 2025-01-25

**Authors:** Somayeh Tajik, Hadi Beitollahi, Fariba Garkani Nejad, Zahra Dourandish, Samuel Adeloju

**Affiliations:** 1 Research Center of Tropical and Infectious Diseases, Kerman University of Medical Sciences, Kerman, Iran; 2 Environment Department, Institute of Science and High Technology and Environmental Sciences, Graduate University of Advanced Technology, Kerman, Iran; 3 School of Chemistry, Monash University, Clayton, Victoria 3800, Australia

**Keywords:** Screen-printed carbon electrode, carbon nanostructures, two-dimensional metal-organic frameworks, cancer disease, chemotherapy, pharmaceutical compounds

## Abstract

**Background and Purpose:**

Cancer is a serious public health concern, hence the determination of dacarbazine as a significant chemotherapeutic agent is very important.

**Experimental approach:**

In the present work, we use a facile method to synthesize a nanocomposite of multi-walled carbon nanotubes (MWCNTs) and two-dimensional zeolitic imidazolate framework nanosheets (2D ZIF-L NSs). The resulting MWCNTs/2D ZIF-L NSs nanocomposite was characterized by field-emission scanning electron microscopy. The MWCNTs/2D ZIF-L NSs nanocomposite was subsequently used to modify a screen-printed carbon electrode (SPCE) to achieve an electrochemical sensing platform for the detection of dacarbazine.

**Key results:**

From cyclic voltammetric studies, it was found that the MWCNTs/2D ZIF-L NSs nanocomposite modified SPCE provided less anodic peak potential (700 mV) and higher anodic peak current (7.7 μA) for oxidation of dacarbazine when compared to other SPCEs. The MWCNTs/2D ZIF-L NSs/SPCE displayed good performance in the quantitative determination of dacarbazine. Under optimum conditions, the differential pulse voltammetry response exhibited a linear concentration range of 0.01 to 80.0 μM for dacarbazine with a relatively high sensitivity of 0.1384 μA μM^-1^ and an estimated detection limit of 0.004 μM. The MWCNTs/2D ZIF-L NSs/SPCE sensor was also successfully applied to the determination of dacarbazine in injections samples of dacarbazine.

**Conclusion:**

This detection method can be used as a valuable tool in the analysis of pharmaceutical formulations to bring benefits in cancer treatment.

## Introduction

Cancer is a class of illnesses characterized by aberrant cell division and proliferation that can spread to different body parts. One often-used technique for treating various cancer tumors is chemotherapy [1]. In order to eradicate cancer cells or stop their proliferation, strong chemotherapeutic agents are administered as part of the chemotherapy process [2]. Depending on the specific type and stage of the cancer being treated, these drugs may be administered via injection, intravenous infusion, or oral medication treatment. Despite its proven effectiveness in treating cancer and improving patient outcomes, chemotherapy has a number of serious side effects [3]. Among these are gastrointestinal problems, exhaustion, hair loss, nausea, vomiting, and weakened immune systems. Also, the ability of chemotherapy to harm or kill healthy cells in addition to malignant ones is a serious issue. Given the wide range of applications of chemotherapy in the treatment of cancer and the accompanying adverse effects, it is necessary to monitor the concentration of chemotherapeutic drugs in various samples in order to improve efficacy and minimize side effects. A potent chemotherapy drug, dacarbazine, has demonstrated effectiveness in treating a number of cancers, such as childhood solid tumors, lung carcinoma, Hodgkin's lymphoma, soft tissue sarcoma, and malignant melanoma [4]. Dacarbazine belongs to the class of alkylating agents and exerts its mechanism of action by damaging the DNA of cancer cells, preventing their replication and ultimately inducing cell death [5]. Despite its beneficial role in cancer treatments, dacarbazine is associated with significant side effects. Common side effects of dacarbazine include vomiting, nausea, constipation, loss of appetite, and fatigue [6]. Furthermore, it can induce myelosuppression, which leads to a decrease in the number of blood cells, including red blood cells, white blood cells, and platelets [7]. Therefore, there is a need for a simple, sensitive and accurate analytical method to measure dacarbazine levels and provide dosage guidance during the chemotherapy procedure. Several methods have been reported for this purpose, including chromatography, capillary electrophoresis, liquid chromatography coupled with mass spectrometry, and electrochemical methods [8-12].

Electrochemical methods offer unique abilities for the rapid determination of very low concentrations of various substances with high sensitivity and reliability [13-15]. Also, unlike many analytical methods, the instrumentation required for electrochemical methods is relatively simple and cost-effective. In addition, electrochemical methods can achieve in-situ measurements and, thus, enable real-time analysis directly at sampling locations if required. Due to its many benefits for electrochemical sensing applications, screen-printing technology is frequently used to fabricate screen-printed electrodes (SPEs) [16,17]. This technology produces electrodes with precise shapes and sizes by printing conductive inks onto a substrate, usually on a flat surface, resulting in the fabrication of a three-electrode system on the same substrate. The key advantages of the SPEs include good sensitivity, cost-effectiveness, ease of fabrication, and reproducibility. SPEs are also portable and easy to miniaturize, thus offering valuable opportunities for on-site and point-of-care applications. Furthermore, the surface of SPEs can be easily modified to improve their performance. Such modification can be tailored to detect a specific analyte or improve its response characteristics [18-20].

Recent studies have reported more electrochemical sensors that are based on the incorporation of various nanomaterials. These have enabled the development of novel electrochemical nano-sensors that are simple to make and use, with fast response time and very high sensitivity [21,22]. One such material that has gained increasing interest for the fabrication of electrochemical sensors is metal-organic frameworks (MOFs). This class of crystalline and porous materials is made up of metal ions or clusters that are coordinated with organic ligands. A large surface area and diverse pore structures characterize these frameworks. Thus, mixing metal and organic components makes it possible to design and produce MOFs with specific characteristics, such as tunable porosity, high adsorption capacity, and selective guest molecule recognition. These characteristics have led to the recent emergence of MOFs as highly promising candidates for a wide range of applications, including catalysis, gas storage and separation, drug delivery, and sensing applications [23].

A distinct class of MOFs that consist of imidazolate linkers and metal ions, which exhibit similar structures to conventional aluminosilicate zeolites, is zeolitic imidazolate frameworks (ZIFs) [24]. In recent years, 2D MOF nanosheets (NSs), particularly 2D ZIF (ZIF-L), have gained significant interest for various applications. This attention stems from their unique features, including large specific surface area, thin thickness, and abundant exposure of active sites [25]. On the other hand, carbon nanostructures, such as carbon nanotubes (CNTs) and graphene, possess remarkable electrical conductivity, high surface area, and impressive mechanical strength. These attributes make them highly suitable candidates for improving the performance of electrochemical sensors [26,27]. These carbon-based materials can function as conductive additives, reinforcing agents, or platforms for immobilizing sensing elements and, thus, enhancing the sensitivity and stability of the sensors. Consequently, by combining MOFs and carbon nanostructures, the final product can demonstrate the benefits of both substances. The synergistic effects of integrating carbon nanostructures and MOFs have enabled the development of highly sensitive, selective and reliable electrochemical sensors [28,29].

In this study, we investigate the use of a MOF, 2D ZIF-L NSs, and MWCNTs as an MWCNTs/2D ZIF-L NSs nanocomposite to fabricate a robust electrochemical sensor for detecting dacarbazine. It was observed that the developed sensor shows a good ability for the oxidation reaction of dacarbazine. Based on DPV measurements, a good linear relationship (*R*^2^ = 0.9996) was found between current and concentration of dacarbazine in the range of 0.01 to 80.0 μM, and the LOD was 0.04 μM. Due to the multiple advantages of MWCNTs and 2D ZIF-L NSs, the prepared sensor showed great performance for the determination of dacarbazine. In addition, the quantitative detection of dacarbazine achieved satisfactory findings in real samples.

## Experimental

### Materials and reagents

All materials were of analytical reagent grade (with higher purity) and were employed in the present work without further purifications. The phosphate buffer solution (0.1 M) was utilized as the supporting electrolyte, which was prepared using phosphoric acid (85 %) and adjusting its pH at different values was conducted using an aqueous solution of sodium hydroxide.

### Instrumentation

The morphological characterization of MWCNTs/2D ZIF-L NSs nanocomposite by FE-SEM analysis was performed using a field-emission scanning electron microscope (MIRA3, TESCAN, Brno, Czech Republic).

The voltammetric studies, including cyclic voltammetry (CV) and differential pulse voltammetry (DPV), as well as chronoamperometric studies, were conducted with a potentiostat-galvanostat Autolab (PGSTAT 302N, Metrohm, Utrecht, the Netherlands). In this study, the DRP-110 model of SPCE was purchased from Dropsens (Oviedo, Spain). The Metrohm-713 pH meter (Herisau, Switzerland) was employed for pH measurements of phosphate buffers.

### Synthesis of MWCNTs/ZIF-L nanocomposite

The synthesis of MWCNTs/ZIF-L nanocomposite was performed as follows: firstly, 30 mg of carboxylated MWCNTs were dispersed in 20 mL of deionized water by ultrasonication for 60 min. Then, 1 mmol of Zn(NO_3_)_2_.6H_2_O (0.297 g) was added to the suspension of CNTs. Also, 8 mmol of 2-methylimidazole (0.655 g) was dissolved separately in 20 ml of deionized water. The suspension of CNTs containing Zn(NO_3_)_2_ and the aqueous solution of 2-methylimidazole were ultrasonicated separately for 5 min. Subsequently, the 2-methylimidazole solution was added dropwise to the CNTs suspension over 10 min. The resulting suspension was stirred with a magnetic stirrer for 2 h at ambient temperature. After this period, the precipitant formed was collected by centrifugation and rinsed multiple times with deionized water. Finally, the MWCNTs/ZIF-L nanocomposite was obtained after drying at 67 °C for 13 h under vacuum.

### Modification of SPCE using MWCNTs/2D ZIF-L NSs nanocomposite

For modification of SPCE, the synthesized MWCNTs/2D ZIF-L NSs nanocomposite (0.5 mg) was dispersed in 0.5 mL of deionized water through ultrasonication for at least 20 min. Then, 4.0 μL of the dispersed nanocomposite was drop-casted on the surface of the working electrode (WE). After drying at room temperature, the MWCNTs/2D ZIF-L NSs nanocomposite-modified SPCE was prepared.

## Results and discussion

### Characterization of MWCNTs/2D ZIF-L NSs nanocomposite by FE-SEM

**Figure 1.**
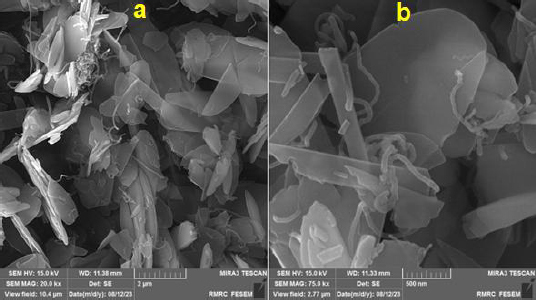
FE-SEM images of MWCNTs/2D ZIF-L NSs nanocomposite at various magnifications (scale bar: 2 μm (a) and scale bar: 500 nm (b))

Figure 1 shows the structure and morphology of the MWCNTs/2D ZIF-L NSs nanocomposite obtained by FE-SEM. The FE-SEM images show the presence of the 2D ZIF-L nanosheets on the MWCNTs surface. Also, 2D ZIF-L nanosheets were intertwined among the MWCNTs.

### Effect of SPCE modification with MWCNTs/2D ZIF-L NSs

**Figure 2.**
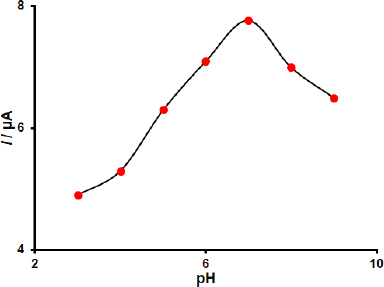
Plot of *I* obtained from DPVs of 50.0 μM dacarbazine *vs.* pH of 0.1 M PBS

The effect of the pH of the phosphate buffer solution (0.1 M PBS) on the detection of dacarbazine with the MWCNTs/2D ZIF-L NSs/SPCE was also investigated by DPV. The pH of PBS was adjusted from pH 3.0 to 9.0. The responses revealed that the most current peak height, *I* of dacarbazine, was achieved at pH 7.0 (Figure 2). Therefore, all further investigations and measurements were conducted in PBS at pH 7.0.

**Figure 3.**
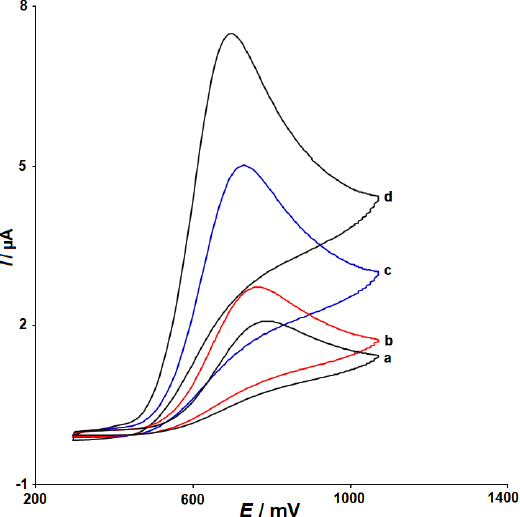
CV responses of unmodified SPCE (voltammogram a), 2D ZIF-L NSs/SPCE (voltammogram b), MWCNTs-COOH/SPCE (voltammogram c), and MWCNTs/2D ZIF-L NSs/SPCE (voltammogram d) in PBS (pH 7.0; 0.1 M) containing 50.0 μM dacarbazine (*ν* = 50 mV s^-1^).

The CV responses obtained for dacarbazine with the unmodified SPCE, 2D ZIF-L NSs modified SPCE, MWCNTs-COOH modified SPCE, and the MWCNTs/2D ZIF-L NSs modified SPCE in PBS (pH 7.0, 0.1 M) are shown in Figure 3. From the recorded CVs, 2D ZIF-L NSs/SPCE demonstrated a higher oxidation peak current (2.7 μA) than unmodified SPCE (2.1 μA), showing the good activity of 2D ZIF-L NSs towards dacarbazine. As for MWCNTs-COOH/SPCE, it demonstrated a much higher oxidation peak current (5 μA) for dacarbazine, revealing the better electrocatalytic performance of MWCNTs-COOH in comparison to 2D ZIF-L NSs. Evidently, the response obtained with the MWCNTs/2D ZIF-L NSs modified SPCE was substantially more sensitive (oxidation peak current = 7.7 μA) than other SPCEs. It proves the efficient role of the simultaneous use of MWCNTs-COOH and 2D ZIF-L NSs in the modification of SPCE towards the oxidation process of dacarbazine. It was also noted that the oxidation peak of dacarbazine shifted toward negative potentials due to the presence of the nanocomposite. The synergistic effect of MWCNTs and 2D ZIF-L NSs was responsible for the observed enhancement of the electrochemical response for dacarbazine.

### Effect of scan rate

**Figure 4.**
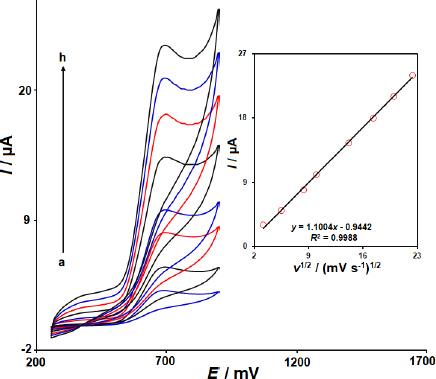
CV responses of MWCNTs/2D ZIF-L NSs/SPCE in 0.1 M PBS (pH 7.0) for electrooxidation of 40.0 μM dacarbazine at scan rates of (a) 10, (b) 30, (c) 70, (d) 100, (e) 200, (f) 300, (g) 400, and (h) 500 mV s^-1^. Inset: The corresponding plot of *I vs. ν* ^1/2^ is shown in the inset

**Figure 5.**
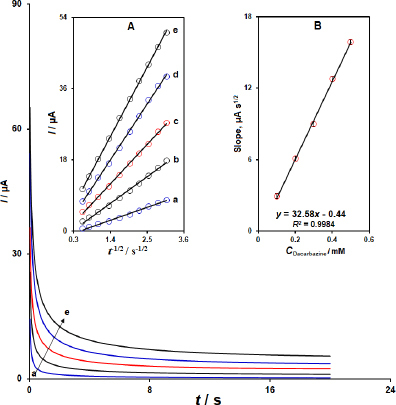
Chronoamperometric response of MWCNTs/2D ZIF-L NSs/SPCE sensor in 0.1 M PBS (pH 7.0) to various concentrations of dacarbazine (chronoamperograms from a to e corresponded to 0.1, 0.2, 0.3, 0.4, and 0.5 mM of dacarbazine, respectively) at the applied potential of 0.75 V versus Ag pseudo-reference electrode. Inset A: Dependence of *I vs. t*^-1/2^ derived from the data of recorded chronoamperograms. Inset B: Linear plot of the slopes from the resulting straight lines versus concentrations of dacarbazine

The CV responses of the MWCNTs/2D ZIF-L NSs/SPCE in PBS (pH 7.0, 0.1 M) to dacarbazine were recorded to evaluate the effect of scan rates ranging from 10 to 500 mV s^-1^ (Figure 4). It was observed that the oxidation currents of dacarbazine increased with an increase in scan rate. Additionally, as presented in Figure 5 (Inset), the oxidation peak currents increased linearly with increasing square root of scan rate ranging from 10 to 500 mV/s, which suggested that the oxidation reaction of dacarbazine was a diffusion-controlled process.

### Chronoamperometric studies

The diffusion coefficient (*D*) of dacarbazine in PBS (pH 7.0) was calculated from chronoamperometric measurements performed with the MWCNTs/2D ZIF-L NSs modified SPCE. The potential of the working electrode (WE) was fixed at 750 mV versus the Ag pseudo-reference electrode, and the chronoamperograms were recorded for various dacarbazine concentrations, as shown in Figure 5.

Then, the plots of *I* versus *t*^-1/2^ were obtained from each recorded chronoamperogram over a certain range of time (Figure 5, Inset A). The mean value of *D* was estimated to be 9.0×10^-5^ cm^2^ s^-1^ from the Cottrell equation: *I* = *nFACD*^1/2^π^-1/2^*t*^-1/2^, where *I* is current response (μA); n is the number of electrons transferred; *F* is Faraday’s constant (96485 C mol^-1^); *t* / s is time; *D* / cm^2^ s^-1^ is diffusion coefficient; and *C* / mol cm^-3^ is the bulk concentration of dacarbazine. The slope value was obtained from the resulting plot of the slopes of the straight lines *vs.* concentrations of dacarbazine (Figure 5, Inset B).

### DPV measurements of dacarbazine at the MWCNTs/2D ZIF-L NSs modified SPCE

**Figure 6.**
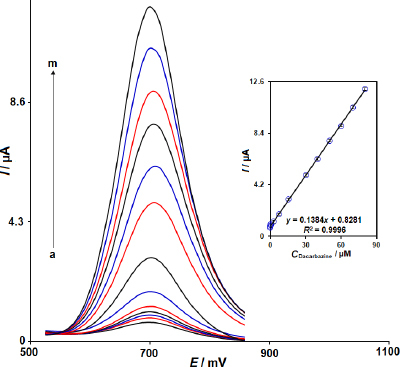
DPV responses of MWCNTs/2D ZIF-L NSs/SPCE in 0.1 M PBS at pH 7.0 for determination of dacarbazine in the concentrations of (a) 0.01, (b) 0.05, (c) 0.1, (d) 0.5, (e) 2.5, (f) 7.5, (g) 15.0, (h) 30.0, (i) 40.0, (j) 50.0, (k) 60.0, (l) 70.0, and (m) 80.0 μM. The inset shows the linear plot of peak current *versus* the concentrations of dacarbazine.

The differential pulse voltammograms obtained for increasing concentrations of dacarbazine in PBS (pH 7.0, 0.1 M) with the MWCNTs/2D ZIF-L NSs/SPCE are shown in Figure 6. Evidently, the anodic peak current increased with increasing dacarbazine concentration and the plot of *I* versus dacarbazine concentration gave a linear relationship from 0.01 μM to 80.0 μM (Figure 6, Inset). The linear relationship was expressed as: *I* = 0.1384*C*_dacarbazine_ + 0.8281 (*R*^2^ = 0.9996)) and the LOD was 0.004 μM for dacarbazine. The LOD was calculated using the formula LOD=3*S*_blank_/*m*, where m is the slope of the linear regression curve and S_b_ is the standard deviation of the response for the blank solution (0.1 M PBS) (obtained based on 12 measurements). The values of LOD and linear range from voltammetric determination in this study are compared with the corresponding values of reported modified electrodes in the literature, which have been used in determining dacarbazine (Table 1). According to Table 1, the values of linear range and LOD of the present work are better or comparable with those reported in the other works.

**Table 1.** Comparative investigation of recent electrochemical sensors reported for determination of dacarbazine.

Modified electrode	Electrochemical method	Linear range, nM	LOD, nM	Ref.
Sn-CeO_2_ nanoparticles modified glassy carbon paste electrode	Square wave voltammetry (SWV)	64 to 6690	3.79	[4]
Sodium dodecyl sulfate (SDS) modified carbon paste electrode (CPE)	DPV	1000 to 4000	150.0	[6]
MWCNTs/CPE	DPV	0.4 to 2500	0.12	[11]
Au nanoparticles-poly (3,4-ethylene dioxythiophene)/reduced graphene oxide nanocomposite modified SPCE	SWV	2.5 to 1450	0.09	[12]
	Chronoamperometry	2.5 to 1675		
MWCNTs/2D ZIF-L NSs/SPCE	DPV	10.0 to 80000.0	4.0	This work

### Stability, reproducibility, and repeatability studies

The stability of the prepared MWCNTs/2D ZIF-L NSs/SPCE sensor towards dacarbazine determination was investigated during the period of 15 days. The DPV measurements of 20.0 μM dacarbazine in 0.1 M PBS at pH 7.0 were conducted every three days using the same electrode kept at ambient temperature. After being kept for 15 days, this sensor still maintained 96.8 % of its initial current response towards dacarbazine.

The investigation of reproducibility was also performed by using DPV in the determination of 30.0 μM dacarbazine using six MWCNTs/2D ZIF-L NSs/SPCEs. These electrodes were prepared in the same way under the same conditions. These prepared SPCEs demonstrated similar voltammetric signals in response to dacarbazine with a relative standard deviation (RSD) of 3.8 %, showing the good reproducibility of the designed sensor.

Furthermore, the repeatability was assessed by repeating the DPV measurement of 20.0 μM dacarbazine in 0.1 M PBS at pH 7.0 (five times) using the same modified electrode (MWCNTs/2D ZIF-L NSs/SPCE). The value of RSD was obtained to be 2.9 %, demonstrating good repeatability of the designed sensor.

### Application of MWCNTs/2D ZIF-L NSs modified SPCE to real sample analysis

To assess the usefulness of the MWCNTs/2D ZIF-L NSs/SPCE for practical applications in real samples, it was applied to the determination of dacarbazine in commercially available dacarbazine injection using a standard addition method. The injection sample was spiked with various concentrations of dacarbazine. Then, the spiked samples were analyzed using the DPV method under optimum conditions. The results obtained from the analysis of the real sample revealed in Table 2 that the sensor’s recoveries for dacarbazine determination in real samples were close to 100 % and the RSDs varied between 1.8 and 3.4 %.

**Table 2.** Application of MWCNTs/2D ZIF-L NSs modified SPCE for determination of dacarbazine in dacarbazine injection

Sample	Concentration, μM		Recovery, %	RSD, % (*n* = 5)
	Spiked	Measured		
Dacarbazine Injection	0	3.3	-	3.4
	2.0	5.5	103.8	2.7
	3.0	6.2	98.4	1.8
	4.0	7.5	102.7	2.9
	5.0	8.0	96.4	`3.0

## Conclusions

In summary, in the present work, we demonstrated the synthesis of MWCNTs/2D ZIF-L NSs nanocomposite using a simple method, which was used to modify the SPCE for electrochemical determination of dacarbazine. The combination of MWCNTs and 2D ZIF-L NSs resulted in a larger surface area and enhanced conductivity, which subsequently enhanced the sensitivity of the oxidation of dacarbazine. The MWCNTs/2D ZIF-L NSs/SPCE sensor successfully achieved a wide range of 0.01 μM to 80.0 μM for dacarbazine with a LOD of 4.0 nM. The electrochemical sensor was also successfully used to determine dacarbazine in injection sample, achieving recoveries ranging from 96.4 to 103.8 %.
